# A Clinical-Radiomics Nomogram Based on Computed Tomography for Predicting Risk of Local Recurrence After Radiotherapy in Nasopharyngeal Carcinoma

**DOI:** 10.3389/fonc.2021.637687

**Published:** 2021-03-18

**Authors:** Chaohua Zhu, Huixian Huang, Xu Liu, Hao Chen, Hailan Jiang, Chaolong Liao, Qiang Pang, Junming Dang, Pei Liu, Heming Lu

**Affiliations:** ^1^Department of Radiation Oncology, People's Hospital of Guangxi Zhuang Autonomous Region, Nanning, China; ^2^Department of Clinical Oncology, Guangxi University of Chinese Medicine, Nanning, China; ^3^Department of Clinical Oncology, Youjiang Medical University of Nationalities, Baise, China

**Keywords:** nasopharyngeal carcinoma, radiomics, prognostic prediction, nomogram, local recurrence

## Abstract

**Purpose:** We aimed to establish a nomogram model based on computed tomography (CT) imaging radiomic signature and clinical factors to predict the risk of local recurrence in nasopharyngeal carcinoma (NPC) after intensity-modulated radiotherapy (IMRT).

**Methods:** This was a retrospective study consisting of 156 NPC patients treated with IMRT. Radiomics features were extracted from the gross tumor volume for nasopharynx (GTVnx) in pretreatment CT images for patients with or without local recurrence. Discriminative radiomics features were selected after *t*-test and the least absolute shrinkage and selection operator (LASSO) analysis. The most stable model was obtained to generate radiomics signature (Rad_Score) by using machine learning models including Logistic Regression, K-Nearest neighbor, Naive Bayes, Decision Tree, Stochastic Gradient Descent, Gradient Booting Tree and Linear Support Vector Classification. A nomogram for local recurrence was established based on Rad_Score and clinical factors. The predictive performance of nomogram was evaluated by discrimination ability and calibration ability. Decision Curve Analysis (DCA) was used to evaluate the clinical benefits of the multi-factor nomogram in predicting local recurrence after IMRT.

**Results:** Local recurrence occurred in 42 patients. A total of 1,452 radiomics features were initially extracted and seven stable features finally selected after LASSO analysis were used for machine learning algorithm modeling to generate Rad_Score. The nomogram showed that the greater Rad_Score was associated with the higher risk of local recurrence. The concordance index, specificity and sensitivity in the training cohort were 0.931 (95%CI:0.8765–0.9856), 91.2 and 82.8%, respectively; whereas, in the validation cohort, they were 0.799 (95%CI: 0.6458–0.9515), 79.4, and 69.2%, respectively.

**Conclusion:** The nomogram based on radiomics signature and clinical factors can predict the risk of local recurrence after IMRT in patients with NPC and provide evidence for early clinical intervention.

## Introduction

Nasopharyngeal carcinoma (NPC) prevails in Southern China. Intensity-modulated radiation therapy (IMRT) is the primary treatment modality for NPC patients and the 5-year survival rate has reached over 80% (1). However, it has been reported that about 10% of patients will eventually experience local recurrence within 3 years after IMRT (2). Therefore, accurate prediction of the risk of local recurrence is crucial to initiate an early individualized intervention (3).

To date, clinical characteristics such as tumor-node-metastasis (TNM) staging, Epstein-Barr virus (EBV) DNA level and tumor volume, are the main prognostic factors for patients with NPC. It has been reported that different responses and survival outcomes may occur after IMRT in patients with the same clinical stage. TNM stage based on anatomy is not always a reliable prognostic factor to precisely predict the recurrence (4). Therefore, it is necessary to find more reliable and practical markers that can reveal tumor heterogeneity before treatment. In recent years, some studies have shown that a large number of molecular markers are associated with tumor growth, metastasis and prognosis in NPC patients (5, 6).It has been acknowledged that with the advances in genomics, genes that drive oncogenesis and disease progression are composed of different genotype subsets. The spatial heterogeneity of solid tumors cannot be revealed by a single invasive biopsy. It has been confirmed that image-based phenotypes can be used to quantify the spatial heterogeneity of tumors (7). Recently, some studies have indicated that radiomics signature generated by using radiomics features which are extracted from high-quality imaging data might be served as imaging markers to predict treatment outcome (8–11).

Radiomics is produced by big data and medical image-assisted diagnosis technology. Radiomics features extracted from region of interest (ROI) in medical images by using a large number of automatic and semi-automatic algorithms can describe in-depth information, such as tumor phenotype and heterogeneity (12, 13). Precise radiotherapy cannot be achieved without medical imaging. CT images contain tumor morphology, texture, and gray level information, which play an important role in target delineation, planning design and dose calculation in radiotherapy for NPC.

Rad_Score is one of the biological markers which reflect the spatial heterogeneity of tumors. In the present study, through data mining method, Rad_Score was generated from radiomics features which were extracted from the gross tumor volume for nasopharynx (GTVnx) in planning CT. We expected that Rad_Score combined with potential clinical factors may help to build and validate a nomogram model for risk prediction of local recurrence in NPC patients.

## Materials and Methods

### Patients

A retrospective analysis was performed for NPC patients who were treated with IMRT in the People's Hospital of Guangxi Zhuang Autonomous Region between January 2016 and December 2017. Eligibility criteria were as the following: histologically proven undifferentiated, non-keratinized carcinoma; after definitive IMRT; stages T1-T4 according to the 2010 AJCC Staging System. Exclusion criteria were as the following: incomplete CT images; lost to follow-up; regional recurrence or distant metastasis after IMRT; death not caused by local recurrence.

### CT Scan

CT images were acquired from a simulation CT scanner (Somatom Sensation Open, Siemens Medical Solutions, Erlangen, Germany) for all patients. Scanning parameters were as follows: 120 kVp, 250 mAs, FOV 500^*^500 mm, 2 mm slice. Fifty-five seconds before CT scan, patients were injected with 95 ml iodixanol. CT image reconstruction was based on filtered back projection (FBP) algorithm.

### Target Delineation and Treatment

The target delineation was in accordance with the International Commission on Radiation Units and Measurements Reports 50 and 62. GTVnx included gross disease determined by CT, MRI or PET-CT. Planning target volume for nasopharnx (PTVnx) was generated by adding 3-mm margin to GTVnx. The plans were designed and optimized using the Pinnacle inverse planning system. The prescribed radiation dose was 70Gy at 2.12 Gy−2.3 Gy per fraction, delivered to PTVnx. All patients were treated once daily, five fractions weekly. Image-guided cone beam computed tomography (CBCT) was performed in all patients before each treatment session.

### CT Radiomics Signature

**Figure 1:**
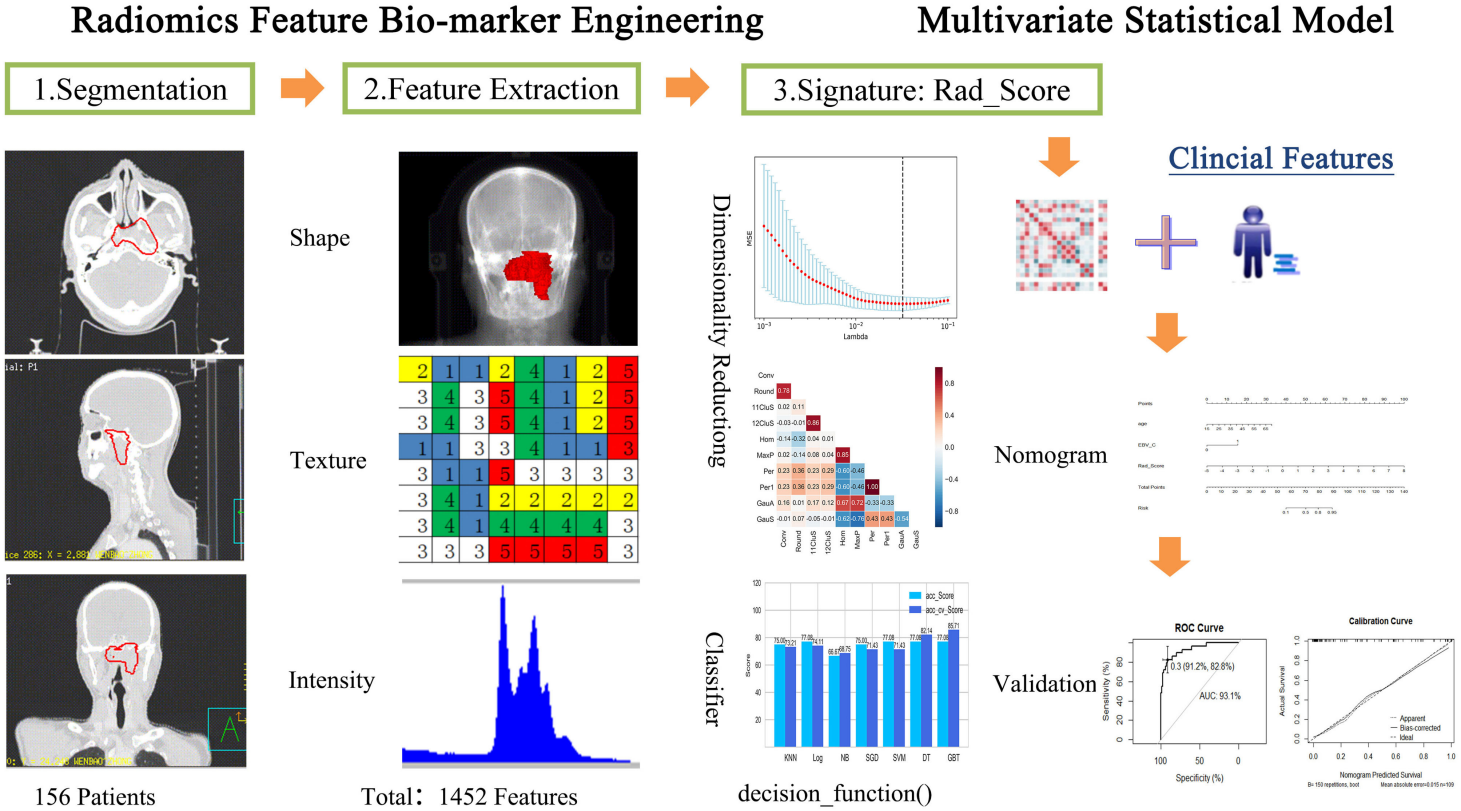
Radiomics processing of the multi-factor model for predicting the risk of local recurrence in NPC patients.

The radiomics workflow is shown in Figure 1: (1) image acquisition and segmentation of ROI; (2) Feature extraction; (3) Feature dimension reduction and Rad_Score generation; (4) Establishment and validation of Nomogram model.

The GTVnx was defined as ROI. Radiomics features were extracted from ROI in IBEX (Imaging Biomarker Explorer, Version 1.0) (14). Radiomics features included Shape, Gray Level Co-occurrence Matrix 25, Gray Level Co-occurrence Matrix 3, Gray Level Run Length Matrix 25, Intensity Direct, Intensity Histogram, Neighbor Intensity 25, Neighbor Intensity Difference 3 and Intensity Histogram Gauss Fit.

The radiomics features extracted from local recurrent and non-recurrent patients were first tested by analysis of variance (ANOVA). The least absolute shrinkage and selection operator (LASSO) algorithm was adopted in selecting the most significant features and feature dimension reduction. The coefficient value of insignificance features was reduced to 0 using 10-fold cross verification.

The selected radiomics features were put into seven types of machine learning classifiers for modeling: K-nearest Neighbor (KNN), Logistic Regression (Log), Naive Bayes (NB), Decision Tree (DT), Stochastic Gradient Descent (SGD), Gradient Boosting Trees (GBT), and Linear Support Vector Classification (SVC).The performance of the predictive models was evaluated by analysis of the area under the curve (AUC) of the receiver operating characteristic (ROC). The most stable classification model was obtained referring to the recall rate and the score of cross-validation. Finally, Rad_Score was generated according to the optimal classification model.

A nomogram was established based on Rad_Score and clinical characteristics including age, gender, T-stage, and changes of EBV-DNA levels in plasma after treatment using logistic regression analysis. Change in EBV-DNA level relative to pre-treatment baseline was considered as a dummy variable and denoted as EBV_C: 1 if EBV-DNA copies remained high or increased compared to its pre-treatment level, otherwise it was 0.

Stepwise method was used to perform variable filtering for simplifying the prediction model, and the optimal nomogram was finally obtained. Predictive performance of the nomogram was evaluated by discrimination and calibration in the training cohort and in the validation cohort. Discrimination was defined as consistency between the predictive ability and the actual recurrence after IMRT based on The Harrell's Concordance Index (C-index). Calibration was defined as consistency between the probability of the outcome predicted by the nomogram and the actual probability. Decision Curve Analysis (DCA) was used to evaluate the clinical benefits of multi-factor nomogram in predicting local recurrence after IMRT.

### Statistical Analyses

The chi-square test for radiomics features was performed using the python 3.7 software package SciPy. Feature dimension reduction and feature modeling were established by LASSO algorithm using the Sklearn package. Multi-factor nomogram was analyzed using the “RMS” and “RMDA” package running in R software, version 4.0.2. For all statistical tests, a probability value (p) of <0.05 was considered statistically significant.

## Results

### Patient Characteristics

One hundred and fifty-six patients were enrolled into either the training cohort or the validation cohort in a ratio of ~2:1 (109:47). There were no significant differences in age, gender, T-stage, or EBV_C between the training cohort and the validation cohort. The patient characteristics included age, gender, T-stage, and plasma EBV_C. The patient characteristics are listed in Table 1.

**Table 1:** Patient characteristics.

**Characteristics**	**Training cohort**	**Validation cohort**	***p***
Age (y, mean ± SD)	46.49 ± 10.62	48.38 ± 10.21	0.165
Sex			0.423
Female	39	13	
Male	70	34	
T stage			0.566
T1	9	5	
T2	30	14	
T3	45	14	
T4	25	14	
EBV_C			0.977
1	89	39	
0	20	8	
Rad_Score, median (interquartile range)	−0.39 (−4.75 to 7.53)	−0.61 (−3.73 to 4.58)	0.651

### Feature Extraction and Selection

**Figure 2:**
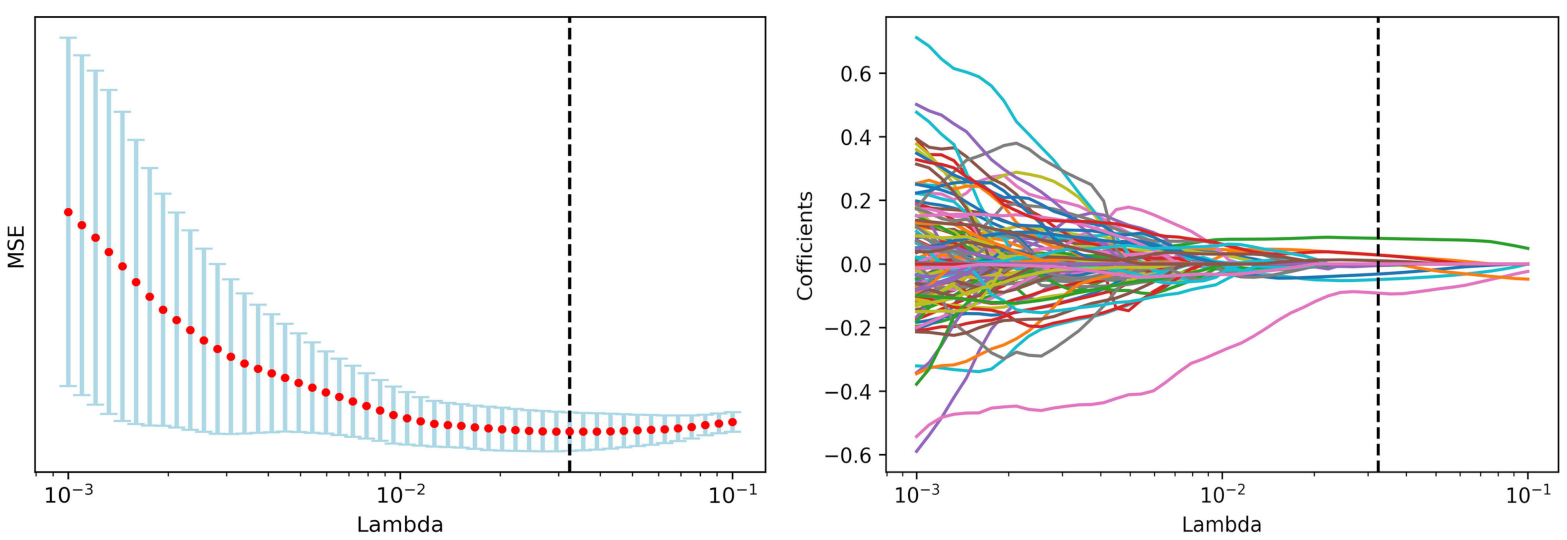
LASSO dimension reduction and Lambda value.

A total of 1,452 radiomics features were initially extracted from ROI in IBEX and 1,027 features with significant differences between local recurrence and non-recurrence patients were finally selected using ANOVA. The loss function reached the minimum value when Lambda is 0.032375 adopting LASSO dimension reduction (15, 16) (Figure 2).

**Figure 3:**
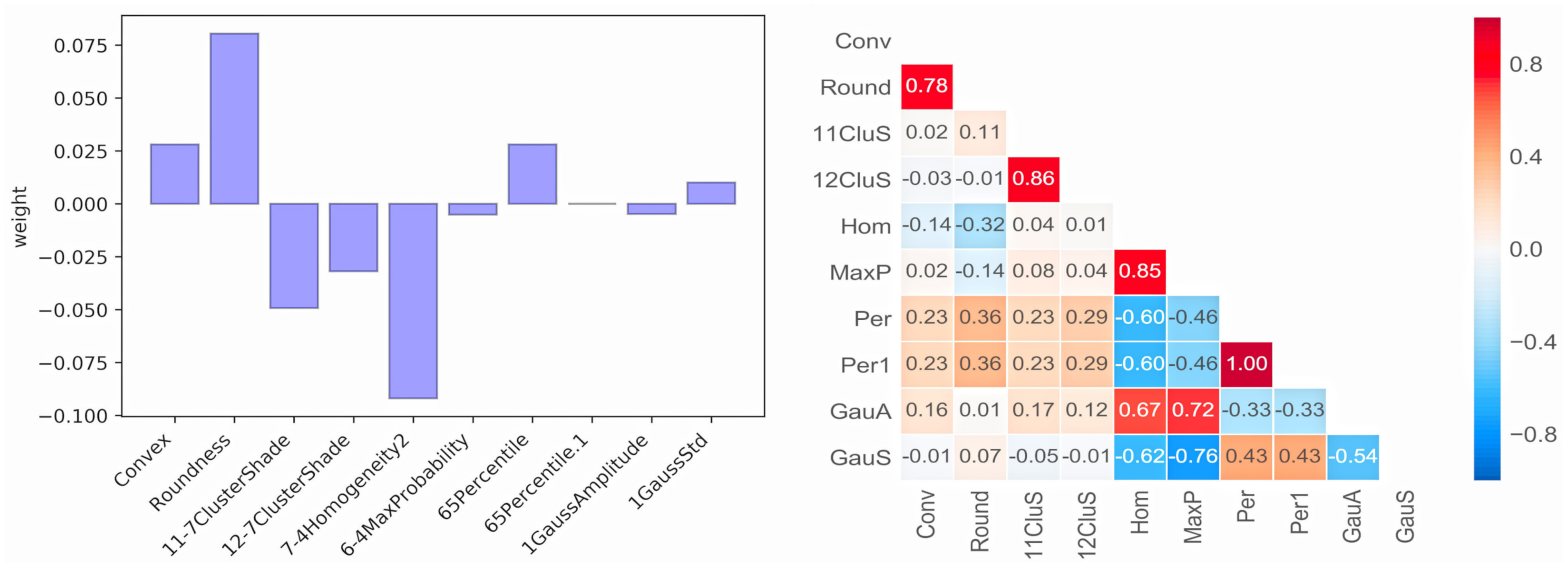
Weight and correlation test of radiomics features.

Ten radiomics features were selected and classified as follows: Shape (Convex, Conv; Roundness, Round), GrayLevelCooccurenceMatrix (11-7ClusterShade, 11CluS; 12-7ClusterShade, 12CluS; 7-4 Homogeneity2, Hom; 6-4MaxProbability, MaxP), IntensityDirect (65Percentile, Per; 65Percentile1, Per1) and IntensityHistogramGaussFit (1GaussAmplitude, GaussA; 1GaussStd, GaussS). The correlation test of 10 features is shown in Figure 3. One radiomics feature was selected from those with a correlation coefficient >0.8, and finally, seven stable selected features (Conv, Round, 11CluS, Hom, Per, GaussA, GaussS) were used for machine learning algorithm modeling (Figure 3).

**Figure 4:**
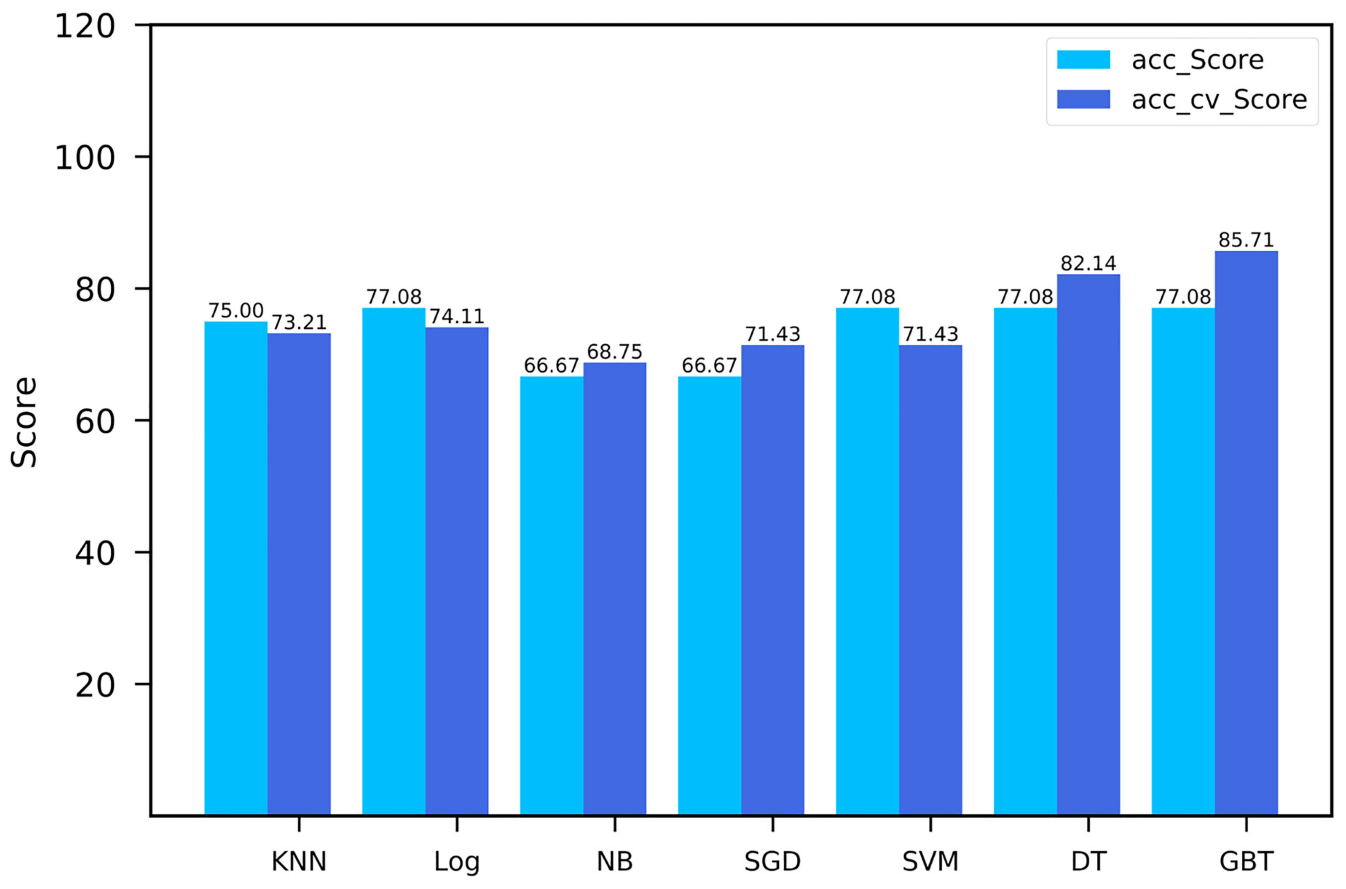
Comparison of seven machine learning models.

The performance comparison of seven machine learning classifiers for modeling on the training cohort is shown in Figure 4. The GBT model got the highest cross-validation score of 85.71, with an AUC of the ROC curve was 0.9 in the validation cohort. Finally, Rad_Score which was defined as the distance from the coordinate point composed of radiomics features to the separating hyperplane was generated according to the GBT model. The training cohort and validation cohort met the requirement of independence as the *P*-value of the chi-square test for Rad_Score was >0.05 (Table 1).

### Nomogram Establishment

**Figure 5:**
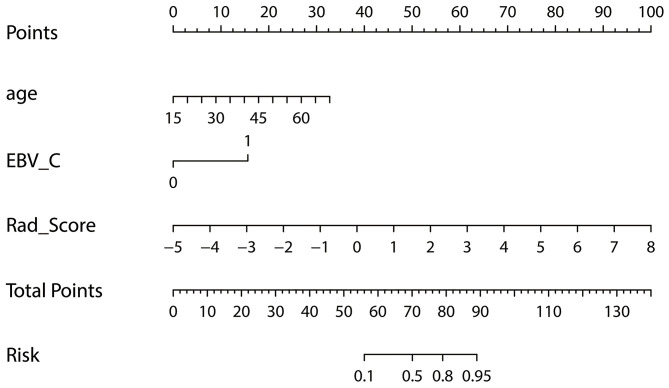
Nomogram for risk prediction of local recurrence in NPC patients after IMRT; Features contained in the training cohort are age, EBV_C and Rad_Score.

A nomogram of local recurrence was finally obtained based on Rad_Score and clinical factors, including age, gender, T-stage, and EBV_C using logistic regression analysis. The differences in age, EBV_C and Rad_Score were statistically significant according to the correlation analysis of the selected variables (Table 2). The actual scores with different variables corresponded to the above point coordinate. Age and Rad_Score were read out with specific values. It got 15 score if the plasma EBV-DNA level increased after IMRT. The sum of the scores of all variables was the total points, and the corresponding risk graph could predict the risk of local recurrence in patients with NPC (Figure 5).

**Table 2:** Multivariable logistic regression.

**Variable**	**Estimate**	**Std. Error**	***z*-value**	***P***
Intercept	−8.17	2.457	−3.325	0.0008^*^
Gender	0.801	0.704	1.138	0.255
Age	0.089	0.039	2.273	0.023^*^
T_stage	0.514	0.413	1.243	0.213
EBV_C	2.529	0.816	3.099	0.0019^*^
Rad_Score	1.148	0.306	3.747	0.000179^*^

* *P < 0.05*.

### Nomogram Validation

**Figure 6:**
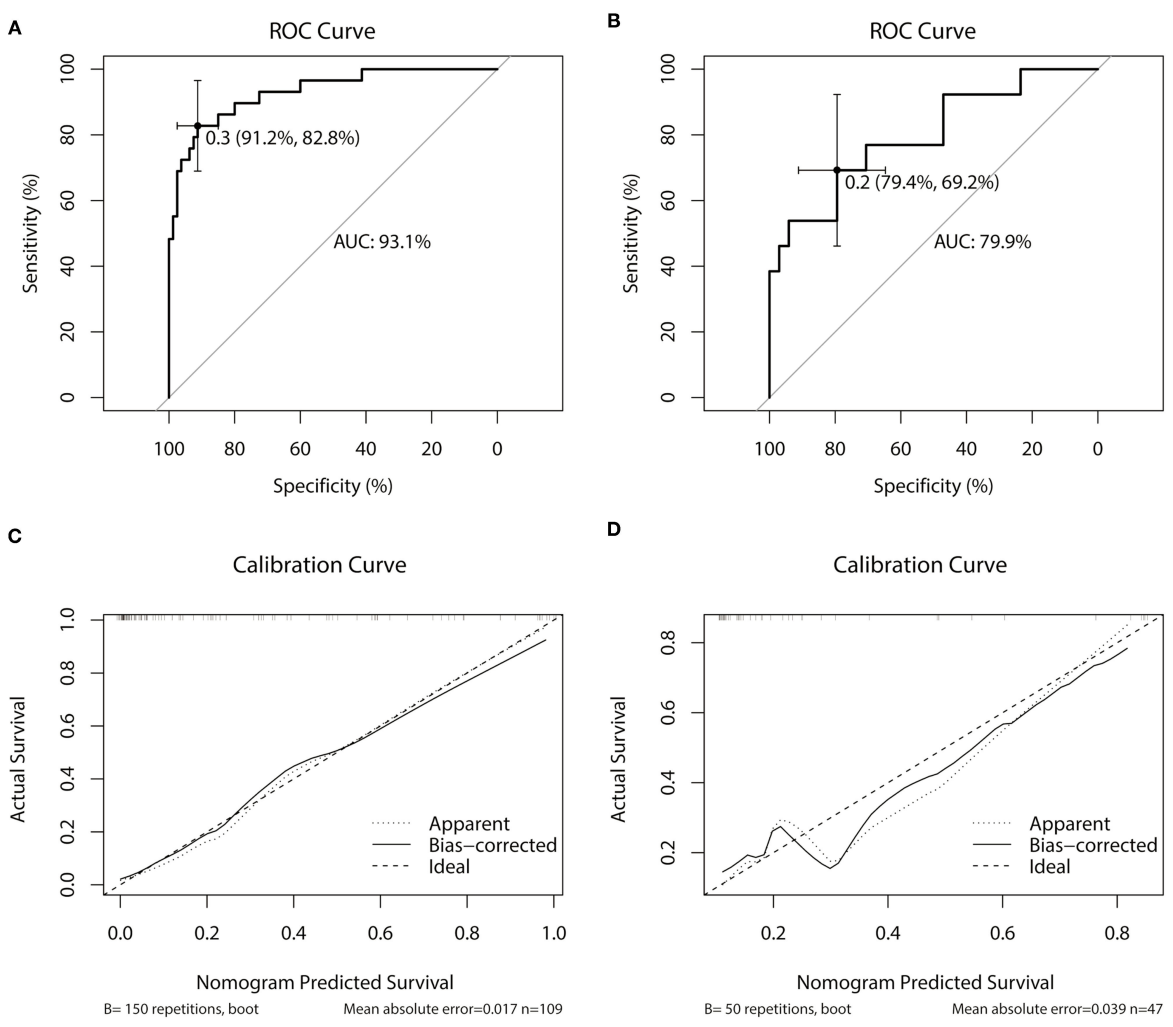
Nomogram validation: **(A)** discrimination curve in the training cohort; **(B)** discrimination curve in the validation cohort; **(C)** calibration curve in the training cohort; **(D)** calibration curve in the training cohort.

The concordance index, specificity and sensitivity in the training cohort were 0.931 (95%CI: 0.8765–0.9856), 91.2 and 82.8%, respectively; whereas, in the validation cohort, they were 0.799 (95%CI: 0.6458–0.9515), 79.4 and 69.2%, respectively (Figures 6A,B).

The calibration curves in the training cohort and the validation cohort are shown in Figures 6C,D. The prediction model showed that the probability distribution of the validation cohort was similar to that of the training cohort. In the validation cohort, when the predicted probability was <0.3, the risk of recurrence was underestimated, and when the predicted probability was >0.3, the risk of recurrence was overestimated.

### Clinical Benefits of Nomogram

**Figure 7:**
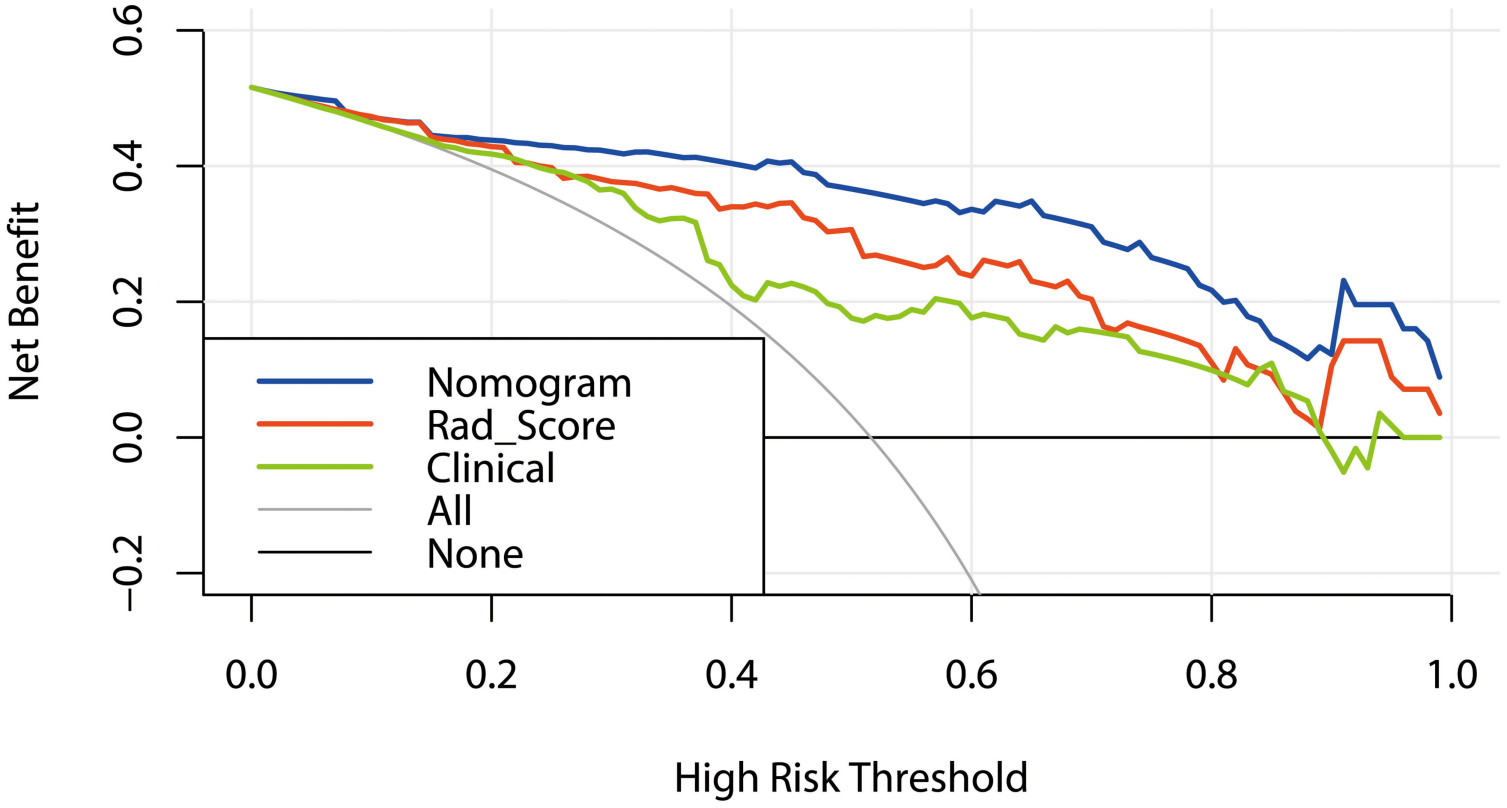
DCA curves: Decision Curve Analysis (DCA). The Y-axis represents net benefit obtained by calculating the difference between the expected clinical benefit and the expected harm in the model. Net Benefit = true positive rate – [false positive rate × weighting factors],weighting factors = threshold probability/(1 – threshold probability). The gray line indicates no local recurrence and the black line indicates local recurrence.

In the training cohort, DCA was used to evaluate the differences in clinical benefits of Rad_Score, clinical factors and nomogram of predicting local recurrence in NPC patients. The DCA curve showed that Rad_Score and Nomogram were better predictors for local recurrence at any given threshold probability than clinical characteristics alone (Figure 7).

## Discussion

It has been one of the major interests for clinicians to provide evidence for early intervention based on the establishment of prognostic prediction model for patients with NPC. Clinical factors selected for predicting outcome vary with time periods and research fields. In this study, Rad_Score was calculated using radiomics signatures extracted from GTVnx of CT images in NPC patients. A multi-factor logistic regression prediction model was built based on clinical factors and Rad_Score to evaluate the risk of local recurrence after IMRT.

Vallières et al. (17) extracted 1,615 radiomics features (quantifying tumor image intensity, shape and texture) and established a prognostic prediction model to evaluate local recurrence and distant metastasis by performing analysis of FDG-PET and CT images in 300 patients with head-and-neck cancer. The survival analysis found that the model was specific for predicting tumor recurrence and metastasis (local recurrence: AUC = 0.69; distant metastasis: AUC = 0.86). Zhang et al. (18) explored the potential of radiomics features based on MRI images in predicting disease progression in patients with advanced NPC. In this study, 970 initial features were extracted from MRI images of 118 patients, and eight features were found to be related to disease progression through dimension reduction analysis. In the present study, we acquired seven radiomics features from GTVnx on simulation CT after two-step dimension reduction. These radiomics features can describe tumor shape, texture, histogram intensity. In order to obtain Rad_Score with strong generalization ability, it is necessary to select a more appropriate machine learning model for specific conditions. Different from the commonly used LASSO, we put the selected radiomic features into seven types of machine learning classifiers for modeling and calculated the Rad_Score of each patient with the most stable classification model.

It is recognized that MRI have high resolution for soft tissue, and fusion of MRI and CT images are often recommended for the delineation of tumor target in radiotherapy. In the present study, we also fused MRI with CT images to delineate the tumor target. Thus, the acquired radiomic features (Convex and Roundness) also contained information about the shape features in MRI images. Zhang et al. (19) established nomogram of local recurrence risk prediction using MRI images and clinical characteristics (gender, age, hemoglobin, N-stage) before treatment. The C-index of the model was 0.74 (95%CI: 0.58–0.85). This was consistent with our findings. The C-index of the validation cohort in our study was 0.799 (95%CI: 0.646–0.952). The possible reasons for the slight difference are due to different image machines, different type of machine learning classifiers for modeling and different selection of clinical characteristics.

There have been a number of studies on multi-factor analysis of clinical factors to predict prognosis of NPC. Tang et al. (20) established a nomogram for predicting disease-free survival based on age, gender, body mass index (BMI), T-stage, N-stage, C-reactive protein, Lactate Dehydrogenase (LDH), and plasma EBV-DNA levels. Sun et al. (21) used age, N-stage, number of lesions, site of metastasis and plasma EBV-DNA level to establish a nomogram predicting metastasis and survival. It has been reported that plasma EBV-DNA level was an important biomarker for predicting the prognosis of NPC patients (22, 23). Plasma EBV-DNA level remained low or negative in clinical remission condition after treatment, but would rise again when recurrence or metastasis occurred. Li et al. (24) found that the plasma EBV-DNA level in local recurrent NPC patients was positive by 51–67%. Huang showed that age was an important factor for predicting prognosis of NPC (25). In the present study, the clinical factors included age, gender, T-stage, and plasma EBV-DNA levels. Similar to the above-mentioned results, we found that age and plasma EBV_C were significantly correlated with local recurrence. However, there was no significant correlation between T-stage and local recurrence. Jiang et al. (26) reported that, although NPC patients with T4 stage may have a high recurrence rate, there was no difference in the local control rate.

Nomogram has been widely used as predictive models in studies about cancer prognosis (27). It can comprehensively analyze the predictive ability of various factors to meet the modeling requirements based on biomarkers and clinical characteristics, so as to achieve personalized therapy. In this study, nomogram established by Rad_Score and some clinical factors could well-describe the heterogeneity of NPC. It could eliminate the need for re-delineation of GTVnx and avoid inter-observer errors because the radiomics features were extracted from the radiotherapy planning. In addition, the shape features of GTVnx contained MRI image information. The validated nomogram found that both the training cohort and the validation cohort had a high degree of discrimination. In the validation cohort, there was a difference in the prediction probability in the calibration, which was possibly attributed to the small sample size and the small positive sample size. The DCA curve showed the comparison of clinical benefit in predicting the risk of local recurrence between clinical characteristics, Rad_Score and nomogram, and the nomogram was found to have better predictive performance than the other two. However, it should be noted that nomogram's predictive performance varies with different clinical treatment models in practical applications.

Our study had some limitations. First, the extracted radiomics features were mainly texture and histogram features of CT and shape features of MRI. The spatial heterogeneity of tumors may be more precisely presented if functional images like functional magnetic resonance image (fMRI) or PET are integrated. Second, the predictive model might perform better if current radiomics which was based solely on spatial heterogeneity of solid tumors is combined with genomic. Finally, independent sample test was conducted in a single institution in this study. Multi-center, large-sample size studies are needed to establish nomogram in more comprehensive clinical settings.

## Conclusion

In conclusion, the nomogram based on CT radiomics signature can predict the risk of local recurrence after IMRT in patients with NPC and provide evidence for early clinical intervention.

## Data Availability Statement

The raw data supporting the conclusions of this article will be made available by the authors, without undue reservation.

## Ethics Statement

The studies involving human participants were reviewed and approved by the Institutional Review Board of the People's Hospital of Guangxi Zhuang Autonomous Region. The patients/participants provided their written informed consent to participate in this study.

## Author Contributions

CZ conceived the project and performed the experiment. HH wrote the paper. XL, HC, HJ, CL, QP, JD, and PL analyzed the data. HL provided expert guidance and reviewed the paper. All the authors gave the final approval of the manuscript.

## Conflict of Interest

The authors declare that the research was conducted in the absence of any commercial or financial relationships that could be construed as a potential conflict of interest.
